# Loss of miR-451a enhances SPARC production during myogenesis

**DOI:** 10.1371/journal.pone.0214301

**Published:** 2019-03-29

**Authors:** Rachel Munk, Jennifer L. Martindale, Xiaoling Yang, Jen-Hao Yang, Ioannis Grammatikakis, Clara Di Germanio, Sarah J. Mitchell, Rafael de Cabo, Elin Lehrmann, Yongqing Zhang, Kevin G. Becker, Vered Raz, Myriam Gorospe, Kotb Abdelmohsen, Amaresh C. Panda

**Affiliations:** 1 Laboratory of Genetics and Genomics, National Institute on Aging, National Institutes of Health, Baltimore, Maryland, United States of America; 2 Translational Gerontology Branch, National Institute on Aging, National Institutes of Health, Baltimore, Maryland, United States of America; 3 Department of Human Genetics, Leiden University Medical Center, Leiden, The Netherlands; Mayo Clinic Minnesota, UNITED STATES

## Abstract

MicroRNAs (miRNAs) are small noncoding RNAs that critically regulate gene expression. Their abundance and function have been linked to a range of physiologic and pathologic processes. In aged monkey muscle, miR-451a and miR-144-3p were far more abundant than in young monkey muscle. This observation led us to hypothesize that miR-451a and miR-144-3p may influence muscle homeostasis. To test if these conserved microRNAs were implicated in myogenesis, we investigated their function in the mouse myoblast line C2C12. The levels of both microRNAs declined with myogenesis; however, only overexpression of miR-451a, but not miR-144-3p, robustly impeded C2C12 differentiation, suggesting an inhibitory role for miR-451a in myogenesis. Further investigation of the regulatory influence of miR-451a identified as one of the major targets *Sparc* mRNA, which encodes a secreted protein acidic and rich in cysteine (SPARC) that functions in wound healing and cellular differentiation. In mouse myoblasts, miR-451a suppressed *Sparc* mRNA translation. Together, our findings indicate that miR-451a is downregulated in differentiated myoblasts and suggest that it decreases C2C12 differentiation at least in part by suppressing SPARC biosynthesis.

## Introduction

Skeletal muscle is a highly specialized tissue necessary for locomotion and energy metabolism in mammals. It is generated through a process known as myogenesis, during which multiple mononucleated myoblasts (satellite cells) are fused to form a multinucleated myofiber, which is the functional unit of the skeletal muscle [1,2]. Myogenesis is regulated transcriptionally by myogenic regulatory factors (MRFs), and post-transcriptionally via RNA-binding proteins (RBPs) and noncoding (nc)RNAs, such as microRNAs (miRNAs) and long noncoding (lnc)RNAs [3–7]. Impairment in the regulation of gene expression during myogenesis can be deleterious, causing atrophy and other muscle pathologies [8].

Mammalian microRNAs (~19–22 nucleotides) generally suppress mRNA translation and/or stability by partial base-pairing with the 3’ untranslated region (UTR) of the target mRNA [9]. This function is carried out through the incorporation of mature microRNAs into the RNA-induced silencing complex (RISC), a large multiprotein complex that contains the microRNA-binding protein argonaute (AGO2) [9]. MicroRNAs have been found to be critical regulators of cellular events such as cell proliferation, differentiation, development, and apoptosis [9]. Accordingly, microRNAs affect tissue and organ function, and dysregulation of microRNAs has been linked to many disease processes including several chronic conditions that increase with advancing age, such as cancer, diabetes, cardiovascular disease, and neurodegeneration [10,11].

To investigate the impact of microRNAs in skeletal muscle as a function of age, we previously surveyed microRNAs in skeletal muscle from young and old Rhesus monkeys [11]. Two microRNAs located on chromosome 17q11.2 and expressed in a cluster, miR-451a and miR-144-3p, were highly upregulated in muscle from older monkeys [11,12]. In this study, we tested the hypothesis that miR-451a/144-3p may be involved in controlling myogenesis. Using the mouse myoblast line C2C12 as a model of myoblast differentiation, our results revealed that miR-451a specifically was downregulated during myogenesis. We further discovered that overexpression of miR-451a suppressed C2C12 myoblast differentiation. Additionally, we identified SPARC (secreted protein acidic and rich in cysteine), an enhancer of myogenesis, as one of the targets of miR-451a. Our findings provide further evidence that miR-451a represses myogenesis at least in part by reducing SPARC production.

## Materials and methods

### Cell culture, transfections and creatine kinase (CK) assay

Mouse C2C12 myoblasts were cultured in growth medium (Dulbecco's Modified Eagle's Medium (DMEM, Life Technologies) containing 20% fetal bovine serum (FBS, Gibco) and antibiotics. Human KM155 myoblasts were cultured in growth medium (equal volume mixture of Hamm’s F10 media with 20% FBS and Promocell Skeletal Muscle Cell Growth Medium). The C2C12 and KM155 myoblasts were maintained at 37°C in a 5% CO_2_ humidified atmosphere and were differentiated by replacing the growth medium with differentiation medium (DMEM with 2% horse serum) [6]. For silencing experiments, control small interfering RNA (Ctrl siRNA) or SPARC siRNA was transfected twice, 36 h and 12 h before inducing differentiation. MicroRNA mimics and inhibitors for miR-451a and miR-144-3p (Sigma Aldrich) were transfected twice, 36 h and 12 h before induction of differentiation at a final concentration of 50 nM using Lipofectamine 2000 (Life Technologies). Creatine kinase (CK) activity was determined in cell lysates using the EnzyChrom creatine kinase assay kit (BioAssay Systems) following the manufacturer's protocol.

### Reverse transcription (RT) followed by real-time quantitative (q) PCR (RT-qPCR) analysis

Total RNA from cultured cells was isolated using TRIzol (Life Technologies) following the manufacturer’s protocol. For cDNA synthesis, reverse transcription (RT) was performed for RNA prepared in TRIzol using Maxima reverse transcriptase following the manufacturer’s protocol (Thermo Fisher Scientific). qPCR analysis of mRNAs was performed according to the manufacturer’s instructions for KAPA SYBR FAST ABI Prism qPCR kit (KAPA Biosystems) with mRNA-specific primers (S1 Table). RT-qPCR reactions were performed on QuantStudio 5 Real-Time PCR System (Thermo Fisher Scientific) with a cycle setup of 2 min at 95°C and 40 cycles of 5 sec at 95°C plus 20 sec at 60°C; the fold change in abundance was calculated as described previously [13].

### Real-time reverse transcription-PCR analysis of miRNAs

Total RNA was reverse-transcribed using the Mir-X microRNA First Strand Synthesis Kit (Clontech) according to the manufacturer's instructions. The microRNAs and siRNAs were quantified by RT-qPCR analysis using KAPA SYBR FAST ABI Prism qPCR kit, miRNA-specific forward primers (S1 Table) and a universal reverse primer. RT-qPCR was performed on QuantStudio 5 Real-Time PCR System (Thermo Fisher Scientific) with a cycle setup consisting of 20 sec at 95°C and 40 cycles of 1 sec at 95°C plus 20 sec at 60°C. The fold change in abundance was calculated using *U6* snRNA as an endogenous normalization control [5].

### Cloning and reporter assays

The 3′UTR of mouse *Sparc* mRNA was amplified with specific primers. After XhoI and NotI double digestion, the PCR product was inserted into the psiCHECK2 plasmid downstream of the Renilla open reading frame (ORF). To create the mutant *Sparc* 3’UTR, we mutated 5 nucleotides in the seed region of the miR-451a target sequence, from TAACG to ATTGC. Mutagenesis was performed using the Site-Directed Mutagenesis Kit (Agilent Technologies #200524–5) and was validated by sequencing. Sixteen hours after co-transfection of miR-451a or Ctrl siRNA with a plasmid (either psiCHECK2, psiCHECK2-Sparc-3′, or psiCHECK2-Sparc-3′mut) using Lipofectamine 2000 in complete medium, cells were lysed and a dual-luciferase assay kit (Promega) was used to measure reporter Renilla luciferase (RL) and firefly luciferase (FL) activity.

### mRNA microarray analysis

Total RNA was labeled according to the manufacturer’s instructions using the Illumina TotalPrepTM RNA amplification kit. A total of 750 ng biotinylated RNA was hybridized overnight to MouseRef-8 v2.0 BeadChip microarrays (Illumina, San Diego, CA). Following post-hybridization rinses, arrays were incubated with streptavidin-conjugated Cy3, and scanned at 0.53 microns using an Illumina iScan scanner. Hybridization intensity data were extracted from the scanned images, and evaluated using Illumina GenomeStudio software, V2011.1. Transcripts with Z-ratio absolute values of ≥1 in both directions were considered to be differentially expressed (S2 Table). The full microarray dataset is available at GSE119186.

### Polysome analysis

Proliferating C2C12 cells were transfected twice, with a 24-h interval, with Ctrl siRNA or miR-451a and cultured for an additional day in growth medium before they were incubated with 100 μg/ml of cycloheximide (Sigma) for 10 min to freeze ribosomes in place on mRNAs. Cytoplasmic lysates were prepared in polysome extraction buffer and fractionated using 10–50% linear sucrose gradients via ultracentrifugation. Twelve fractions were collected for each sample and the RNA in each fraction was isolated using TRIzol (Life Technologies) and quantified by RT-qPCR analysis [14].

### Protein analysis

Total protein lysates were prepared in RIPA buffer containing protease inhibitor. Proteins were size-separated by SDS-PAGE and transferred onto nitrocellulose membrane (Life Technologies). For Western blot analysis, the primary antibodies employed recognized SPARC (Cell Signaling), HSP90 (Santa Cruz Biotechnology) or GAPDH (Santa Cruz Biotechnology). After incubation with appropriate secondary antibodies, protein signals were developed using chemiluminescence. The intensities of the bands on Western blots were quantified using the ImageJ program. The relative expression levels of SPARC protein were calculated after normalizing to the abundance of endogenous loading controls GAPDH or HSP90.

### Statistical analysis

Data are expressed as the means ± SEM unless indicated otherwise. Statistical comparisons of the RT-qPCR results were evaluated using Student's t-test. A P-value of <0.05 was considered statistically significant.

## Results

### miR-451a/144-3p levels correlate with skeletal muscle regeneration *ex vivo*

**Fig 1:**
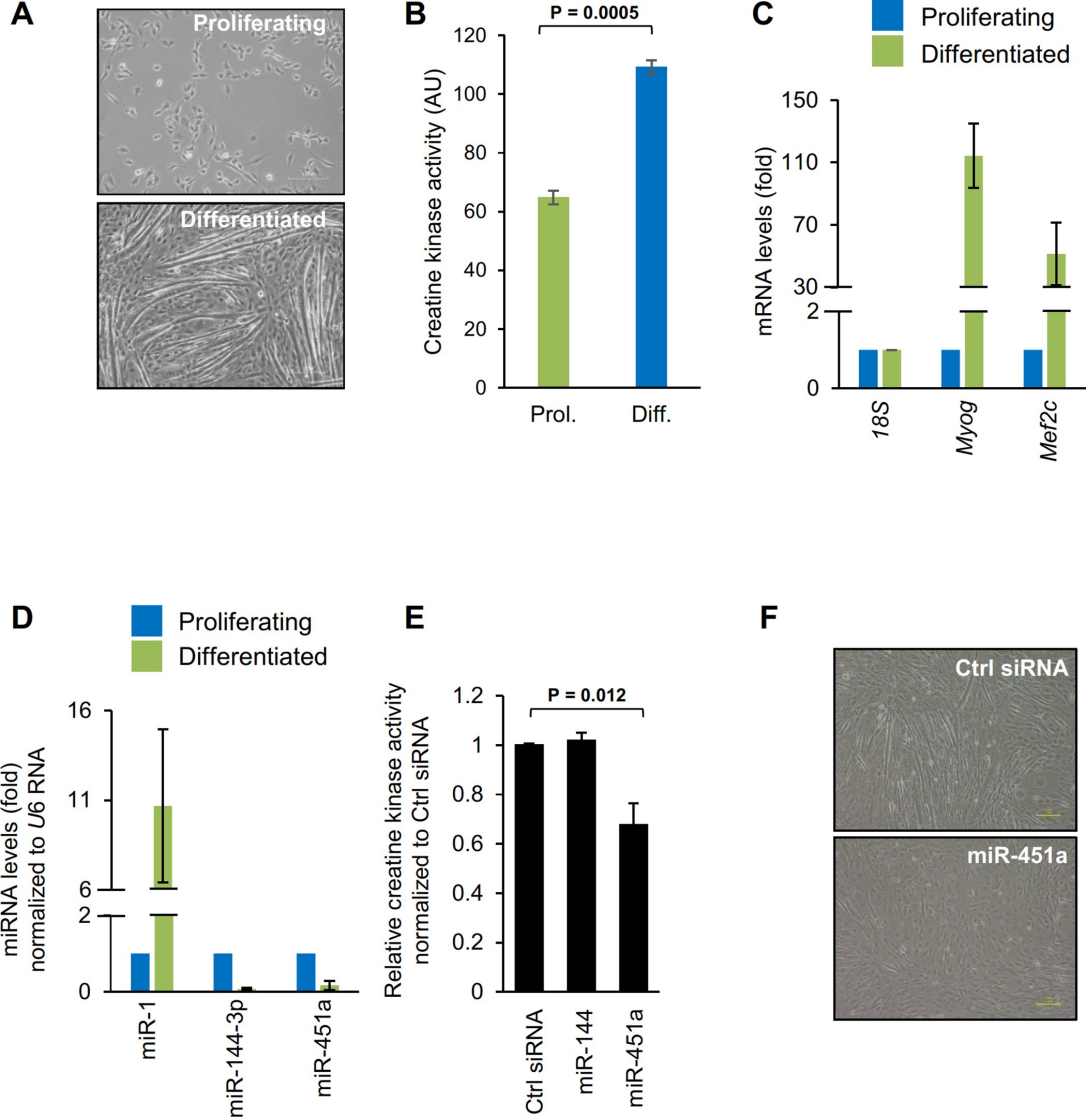
Inhibition of myoblast differentiation by miR-451a. **(A)** Phase-contrast microscopy of C2C12 cells that were cultured in either proliferating medium or cultured for 6 days in differentiating medium. **(B)** Creatine kinase levels in the proliferating C2C12 cells and differentiated myotubes. **(C)** Changes in mRNA levels upon C2C12 myoblast differentiation on day 6 as quantified by RT-qPCR using *18S* rRNA as normalization control. **(D)** RT-qPCR analysis of miR-1, miR-144-3p, and miR-451a in proliferating and differentiated C2C12 myoblasts. **(E)** Analysis of creatine kinase activity in differentiated C2C12 myoblasts transfected with the indicated miRNAs. **(F)** Phase-contrast fields of C2C12 cells transfected with Ctrl siRNA or miR-451a mimic followed by culture for 5 days in differentiation medium. Data in panel A-D represent the means ± SEM from 3 independent experiments. Significance (P) is indicated.

C2C12 myoblasts have been used extensively as a model to study differentiation of skeletal muscle [5–7]. As shown in Fig 1A, replacing the growth medium (GM) of proliferating C2C12 cells with differentiation medium (DM, Materials and Methods) triggered myoblasts to differentiate into multinucleated myotubes resulting from the fusion of myoblasts. Differentiation was further confirmed by measuring the creatine kinase (CK) activity; CK activity was higher in differentiated conditions (Fig 1B) than in proliferating cells, supporting the differentiation into myotubes. As previously reported, *Myog* and *Mef2c* mRNAs, encoding the key differentiation markers MYOG (myogenin) and MEF2C, were dramatically upregulated during myoblast differentiation, displaying highest abundance by day 6 of culture in DM (Fig 1C). RT-qPCR analysis showed that the levels of miR-451a and miR-144-3p declined in myotubes, while the levels of miR-1 (a marker of differentiation) rose considerably (Fig 1D). Analysis of miR-451a levels in KM155 human myoblasts similarly revealed that miR-451a levels were reduced as cells differentiated into myotubes (S1 Fig).

### miR-451a inhibits myoblast differentiation

miR-451a was previously shown to regulate various physiological and pathological processes including cell proliferation, cancer, and erythropoiesis [15–18]. Since the abundance of miR-451a/144-3p declined significantly during C2C12 differentiation, we investigated whether miR-451a/144-3p influenced C2C12 myogenesis. As shown in Fig 1E, overexpression of miR-451a significantly decreased C2C12 differentiation, as assessed by measuring creatine kinase activity, a marker of myogenesis, on differentiation day 6, suggesting that miR-451a inhibits muscle cell differentiation. However, neither the overexpression nor the inhibition of miR-144-3p altered C2C12 differentiation. Phase-contrast microscopy indicated that overexpression of miR-451a, but not miR-144-3p (not shown), reduced substantially the formation of myotubes (Fig 1F). These findings indicate that miR-451a specifically suppressed the differentiation of C2C12 myoblasts.

### miR-451a binds the *Sparc* 3′UTR

**Fig 2:**
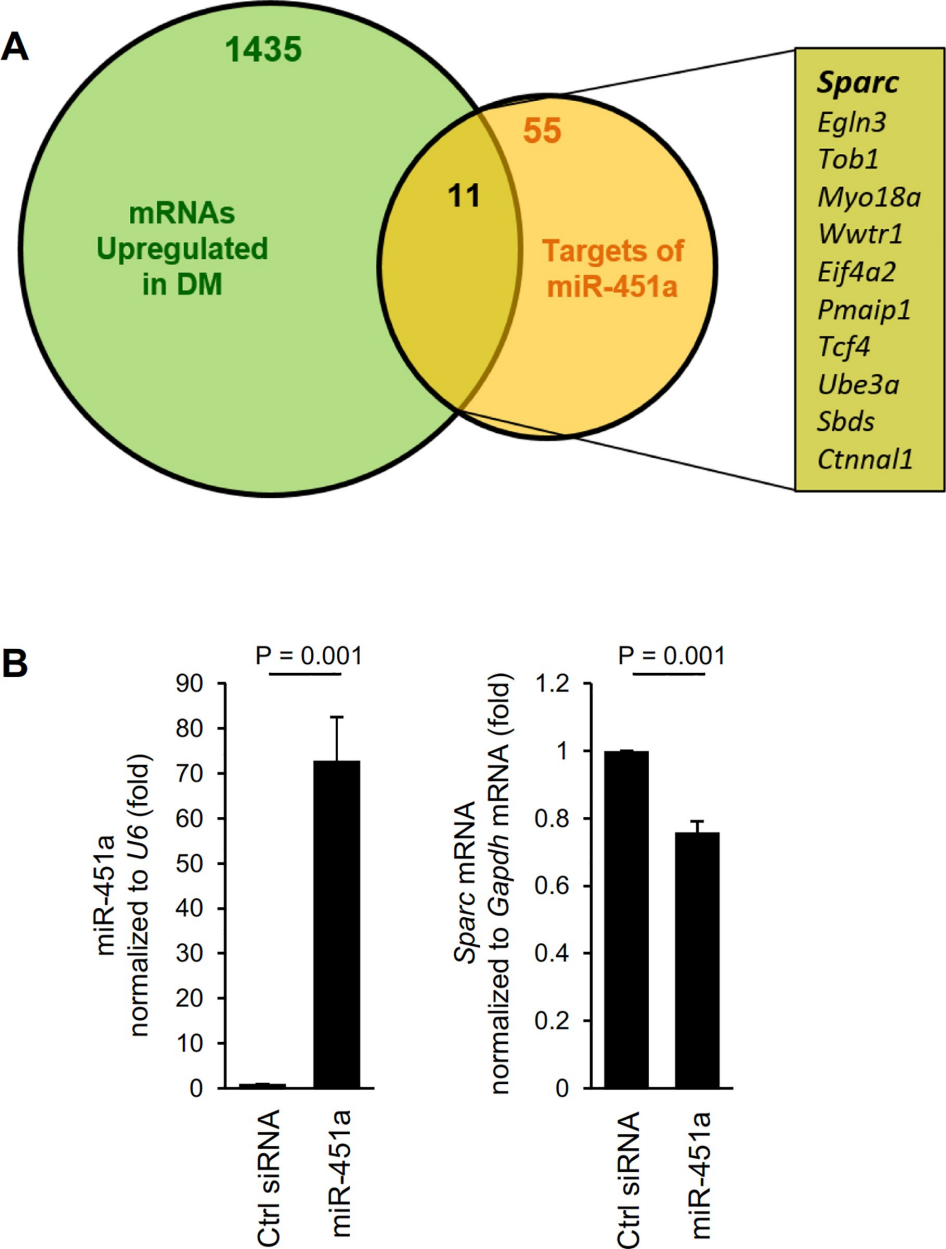
Identification of miR-451a targets in muscle cells. **(A)** Venn diagram representation of mRNAs upregulated during C2C12 differentiation (green) and experimentally identified target mRNAs of miR-451a (orange) to find miR-451a targets implicated in regulating myogenesis. (**B)** RT-qPCR analysis of miR-451a level after overexpression of miR-451a in C2C12 cells (*left*). RT-qPCR analysis of *Sparc* mRNA induction after overexpression of miR-451a in C2C12 cells (*right*). Data represent the means ± SEM from 3 independent experiments. Significance (P) is indicated.

To identify systematically the possible mediator(s) whereby miR-451a influenced C2C12 differentiation, we undertook two strategies: (1) we used microarrays to identify mRNAs known to show increased abundance in C2C12 differentiation (Fig 2A, green; S2 Table) [7], and (2) we identified mRNAs associated with miR-451a reported from AGO2 CLASH dataset (starBase V2.0 http://starbase.sysu.edu.cn/) (Fig 2A, orange, S2A Fig, and S3 Table) [19,20]. Our microarray analysis revealed more than 1400 transcripts differentially expressed (Z-ratio ≥ 1) in differentiating C2C12 cells compared to proliferating myoblasts (S2 Table) (a partial list of mRNAs differentially expressed during C2C12 differentiation was reported earlier [7]). From among the transcripts that were both upregulated in differentiated cells and associated with miR-451a, we sought to identify known factors implicated in regulating myoblast differentiation (Fig 2A). One transcript, *Sparc* mRNA, met all three criteria.

The AGO2 CLASH analysis identified miR-451a as directly interacting with the 3’UTR of *Sparc* mRNA (S2A Fig). To study the effect of miR-451a on SPARC expression in C2C12 cells, we overexpressed either miR-451a or control miRNA (Fig 2B, *left*). Reverse transcription (RT) followed by real-time quantitative (q)PCR analysis indicated that *Sparc* mRNA was moderately downregulated in miR-451a-transfected cells compared to control cells (Fig 2B, *right*). Together, these data indicated that miR-451a suppressed SPARC production in C2C12 cells at least in part by reducing *Sparc* mRNA levels.

### miR-451a inhibits SPARC translation

**Fig 3:**
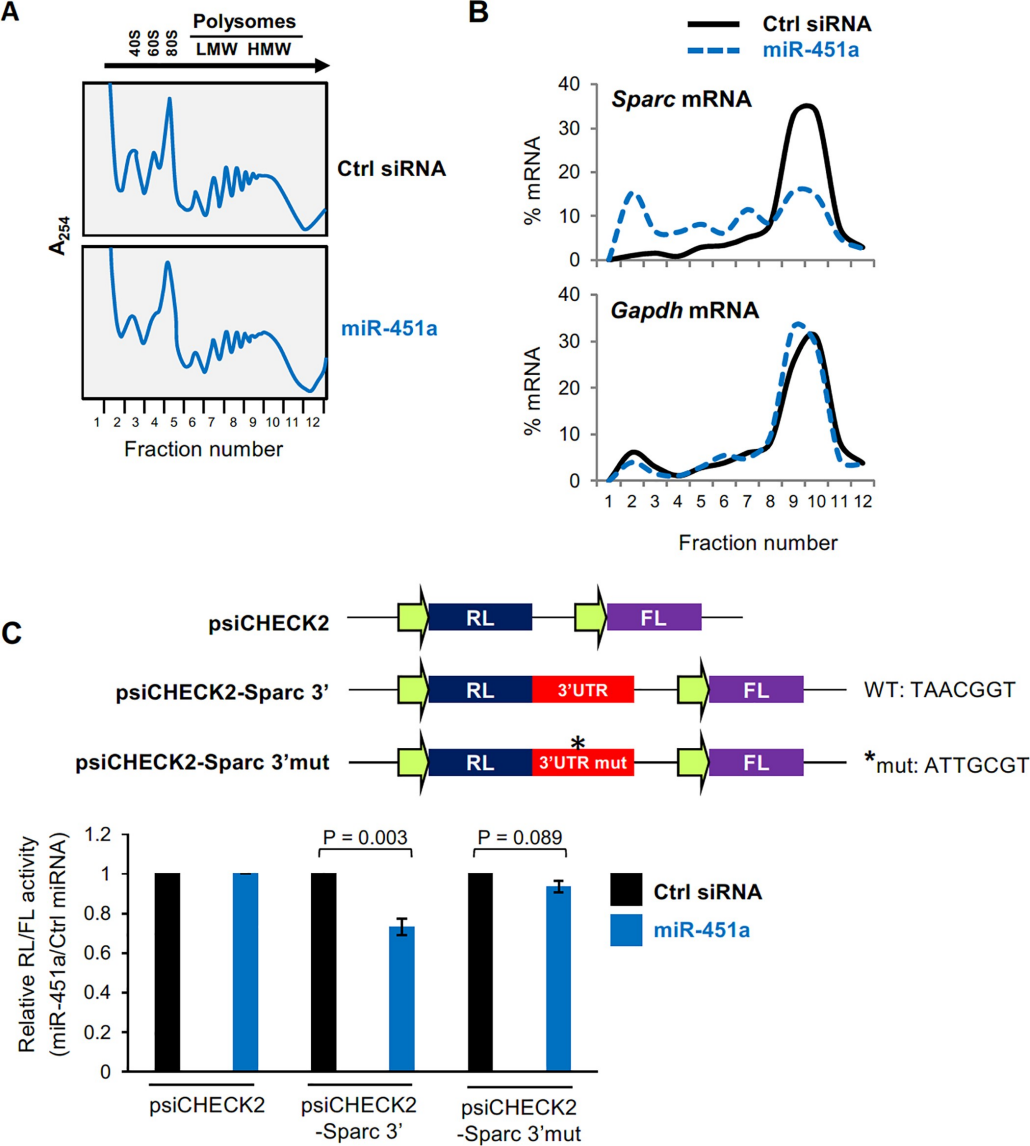
miR-451a inhibits translation of *Sparc* mRNA. **(A)** Global polysome profile of C2C12 cells 48 h after transfection either with Ctrl siRNA or miR-451. **(B)** The relative distribution of *Sparc* mRNA (and housekeeping gene *Gapdh* mRNA) was studied by RT-qPCR analysis of RNA in each of 12 polysome fractions. **(C)** *Top*, schematic of the dual-luciferase reporter plasmids psiCHECK2, the control vector expressing renilla luciferase (RL) and the internal control firefly luciferase (FL); the test vector psiCHECK2-Sparc-3′ contains the *Sparc* 3′UTR downstream of the RL coding region and the psiCHECK2-Sparc-3′-Mut contains the *Sparc* 3′UTR downstream of the RL coding region with mutated target site for miR-451a. *Bottom*, C2C12 cells were transfected with either miR-451a or Ctrl siRNA and either psiCHECK2, psiCHECK2-Sparc 3’ or psiCHECK2-Sparc 3’mut; the ratio of RL activity to FL activity was calculated 16 h after transfection. The relative RL/FL ratios of miR-451a-transfected cells relative to RL/FL of Ctrl siRNA-transfected cells are indicated. Data represent the means ± SEM from 3 independent experiments. Significance (P) is indicated.

To analyze if miR-451a further regulated SPARC production by influencing SPARC translation rate, we quantified the distribution of *Sparc* mRNA present in polysomes from proliferating C2C12 cells expressing normal or ectopically increased levels of miR-451a. Cytoplasmic lysates were fractionated on sucrose gradients, and the relative level of *Sparc* mRNA in each fraction was measured (Fig 3A and 3B). In cells transfected with control (Ctrl) siRNA, *Sparc* mRNA levels were low in the lighter fractions of the gradient (fractions 1–5), which included free RNA and RNA associated with ribosomal subunits (40S and 60S) and monosomes (80S) (Fig 3A), while *Sparc* mRNA levels were high in fractions 9 and 10, which contained the most actively translating polyribosomes (Fig 3B). Importantly, overexpression of miR-451a triggered a leftward shift in the distribution of *Sparc* mRNA on the gradient, indicating a reduction in the size of polysomes associated with *Sparc* mRNA, and suggesting a reduction in *Sparc* mRNA translation. As a control, the distribution of *Gapdh* mRNA, encoding the housekeeping protein GAPDH, was unaffected by miR-451a overexpression.

The effect of miR-451a on *Sparc* mRNA was further assessed using a heterologous luciferase reporter vector (psiCHECK2) bearing the 3′UTR of *Sparc* mRNA (psiCHECK2-Sparc-3′) and a further reporter in which the target sequence of miR-451a was mutated in the *Sparc* 3′UTR (psiCHECK2-Sparc-3′mut) (Fig 3C). C2C12 cells were transfected with Ctrl siRNA or miR-451a along with one of three reporter plasmids–psiCHECK2, psiCHECK2-Sparc-3’ or psiCHECK2-Sparc-3’mut. Sixteen hours later, the ratio of Renilla luciferase (RL), encoded by the reporter transcript bearing the wild type or mutant *Sparc* 3′UTR, to firefly luciferase (FL), encoded by an internal control reporter transcript, was calculated and was set as 1 for the parent vector (psiCHECK2). RL/FL ratios in miR-451a transfected cells were studied relative to RL/FL ratios in cells transfected with control siRNA (Fig 3C). Overexpression of miR-451a repressed luciferase expression from the psiCHECK2-Sparc-3’ reporter but it did not affect the expression of the psiCHECK2-Sparc-3’mut reporter, supporting the hypothesis that miR-451a suppressed SPARC expression by interacting with the *Sparc* 3′UTR. These results indicate that miR-451a represses *Sparc* mRNA translation.

### miR-451a may inhibit myogenesis in part by suppressing SPARC expression

**Fig 4:**
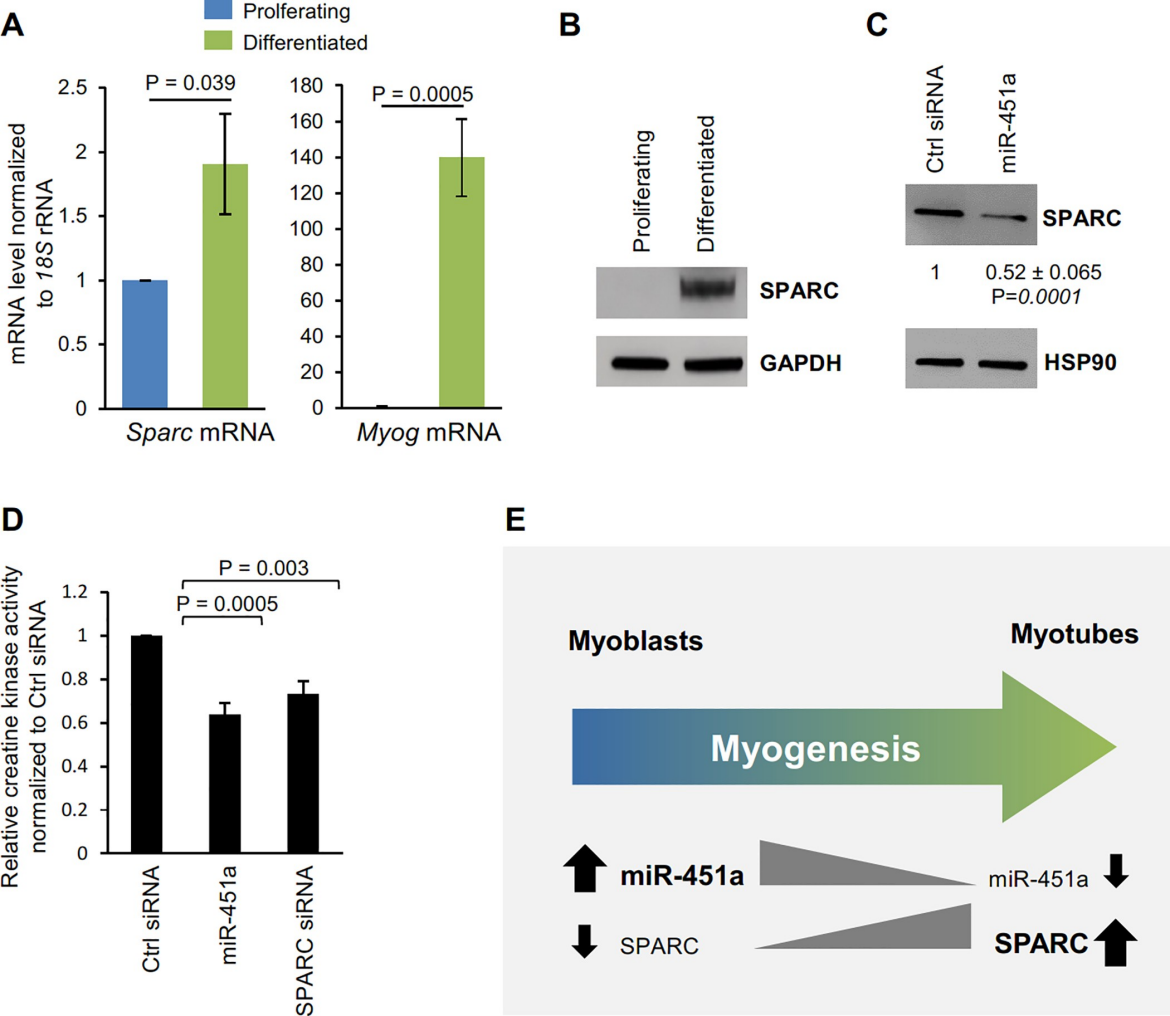
miR-451a inhibits myoblast differentiation by suppressing SPARC expression. **(A)** RT-qPCR analysis of *Sparc* and *Myog* mRNAs during C2C12 cell differentiation. **(B)** Western blot analysis of SPARC in C2C12 cells in proliferating phase and 6 days in differentiated medium. **(C)** Western blot analysis of the levels of SPARC and loading control HSP90 in C2C12 cells transfected with Ctrl siRNA or miR-451a. The relative intensities of the SPARC bands were quantified, normalized to the intensities of the HSP90 bands, and were represented as the means ± SEM. **(D)** Analysis of creatine kinase activity in differentiated C2C12 myoblasts transfected with either Ctrl siRNA, miR-451a or SPARC siRNA. **(E)** Proposed model of miR-451a influencing SPARC expression. miR-451a can inhibit SPARC expression by moderately lowering *Sparc* mRNA levels robustly inhibiting the translation of *Sparc* mRNA. We propose that by inhibiting SPARC biosynthesis, miR-451a suppresses myogenesis. Data in panel A-D represent the means ± SEM from 3–4 independent experiments. Significance (P) is indicated.

Since miR-451a inhibits the expression of SPARC in C2C12 cells, we investigated whether SPARC expression was altered during C2C12 differentiation. As shown in Fig 4A and 4B, *Sparc* mRNA and protein levels are significantly upregulated during C2C12 differentiation. Importantly, the increase in *Sparc* mRNA levels correlated inversely with the levels of miR-451a (Fig 1D) and, as expected, overexpression of miR-451a in C2C12 cells decreased SPARC expression (Fig 4C). Furthermore, the levels of *SPARC* mRNA and SPARC protein were significantly upregulated while the levels of miR-451a was downregulated during KM155 human myoblast differentiation (S1 Fig). SPARC silencing and miR-451a overexpression decreased myogenesis by day 6, as assessed by measuring the activity of the differentiation marker creatine kinase (Figs 1E and 4D). Taken together, these findings indicate that miR-451a may inhibits myogenesis at least in part by suppressing the levels of SPARC in C2C12 myoblasts, as shown schematically in Fig 4E.

## Discussion

The differentiation of myoblasts into myotubes and muscle is accompanied by several steps including an initial burst of proliferation of myoblasts, followed by myoblast elongation, and fusion of elongated myotubes. This morphological remodeling of myoblasts to myotubes is carried out by numerous growth factors, cytokines, cytoskeletal proteins, and extracellular matrix proteins [21,22]. Studies conducted in different cell types have shown that SPARC, an extracellular Ca^2+^-binding glycoprotein, is involved in cell adhesion, differentiation, wound healing, angiogenesis, adipogenesis, and growth factor-binding [23,24]. SPARC is also upregulated during C2C12 and MM14 myoblast differentiation and promotes myogenesis [25]. A recent study reported that SPARC expression declines in old skeletal muscle [26]. Silencing of SPARC in skeletal muscle leads to increased expression of atrogin1 and TGFβ signaling, which in turn decreases the diameter of fast-type myofibers [26].

We previously reported that several miRNAs were altered during skeletal muscle aging and that miR-451a and miR-144-3p in particular were highly elevated with aging [11]. Interestingly, while miR-451a and miR-144-3p are generated from the same parental bicistronic precursor miRNA transcript [27], the maturation of miR-451a is independent of DICER and follows a noncanonical miRNA biogenesis pathway requiring DROSHA and AGO2 [12,28]. miR-451a is highly conserved among vertebrates and is abundant in erythropoietic cells [12,14], rising during erythropoiesis and acting as a positive regulator of erythroid maturation [14,29]. Moreover, miR-451a is dysregulated in many human cancers and promotes carcinogenesis [30]. Interestingly, miRNA profiling in proliferating and differentiated human purified CD56+ myoblasts revealed that miR-451 was downregulated during human myoblast differentiation [31]. Additionally, miR-451 levels increased in individuals exhibiting a low response to resistance exercise training, an intervention that stimulates muscle growth by increasing the production of proteins important for remodeling muscle and augmenting muscle size [32]. The upregulation of miR-451 in low responders who fail to increase muscle mass upon resistance exercise suggest an inhibitory effect of miR-451 on muscle growth *in vivo* [32]. At the same time, miR-144 was shown to regulate the expression of α-hemoglobin in zebrafish by modulating the levels of the transcription factor KLFD [33].

In the present study, we investigated the role of miR-451a and miR-144-3p using C2C12 myoblasts, a culture cell model of skeletal myogenesis. Interestingly, expression of both miR-451a and miR-144-3p decreased during C2C12 differentiation (Fig 1D), but only overexpression of miR-451a inhibited muscle differentiation, while miR-144-3p had no effect at all. To identify the targets of miR-451a in muscle cells, we surveyed mRNAs differentially expressed during C2C12 myoblast differentiation and mRNAs predicted by AGO2 CLASH to be targets of miR-451a. These criteria identified SPARC as a miR-451a-regulated protein in myoblasts (Fig 2, S2A and S2B Fig). As anticipated, SPARC expression levels increased in differentiated C2C12 cells, coinciding with miR-451a decline. In keeping with the findings that overexpressing miR-451a in C2C12 cells suppressed SPARC production but affected only modestly *Sparc* mRNA levels, and that *Sparc* mRNA associated with smaller, less translating polysomes when miR-451a was antagonized, we propose that miR-451a primarily reduced the translation of *Sparc* mRNA. In addition, miR-451a suppressed the expression of a reporter construct bearing the 3’UTR of *Sparc* mRNA, indicating that the 3’UTR was responsible for mediating the translational regulation of SPARC by miR-451a (Fig 3C).

Based on the findings that silencing SPARC or overexpressing miR-451a both inhibited muscle differentiation, we further hypothesize that the regulation of muscle differentiation by miR-451a could be through regulating the expression of SPARC (Fig 4). Extensive attempts at rescuing the inhibition of miR-451a with recombinant SPARC, or with ectopically delivered plasmid and viral SPARC expression vectors were inconclusive (not shown). Perhaps functional modifications are needed and/or the range of functional SPARC overexpression is very narrow [34]. Interestingly, in aging mouse skeletal muscle, miR-451a expression rises while *Sparc* mRNA expression declines with age (S2A and S2B Fig). This suggests that the downregulation of SPARC in old skeletal muscle could be due to upregulation of miR-451a (S3C Fig). Collectively, our findings suggest that the age-associated decline in muscle regeneration may be linked to the rise in miR-451a abundance with age, leading to suppression of SPARC production. We propose that interventions to modulate miR-451a levels may benefit muscle function therapeutically.

## Supporting information

S1 Materials and Methods Supporting method pertaining to S1 Fig. (DOCX)

S1 Fig Expression levels of miR-451a and SPARC in differentiating KM155 human myoblast cell line. **(A)** Phase-contrast micrographs of KM155 cells that were cultured in either proliferating medium or cultured for 6 days in differentiating medium at 37°C in a 5% CO_2_ humidified atmosphere. **(B)** The cultures described in (A) were used for RT-qPCR analysis of *SPARC* mRNA and miR-451a in proliferating or differentiated KM155 cultures. Data represent the means ± SEM from 3 or 4 independent experiments. Significance (P) is indicated. **(C)** Western blot analysis of MYH and SPARC levels in the KM155 cultures described in panel (A).
(PDF)

S2 Fig Identification of miR-451a as a microRNA interacting with *Sparc* mRNA. **(A)** AGO HITS-CLIP data reported by starBase V2.0 (http://starbase.sysu.edu.cn/). **(B)** Schematic representation of miR-451a target site in the 3’UTR of mouse *Sparc* mRNA as predicted by microrna.org that uses the miRanda algorithm for target prediction.
(PDF)

S3 Fig Age-associated changes in the levels of miR-451a and *Sparc* mRNA in mouse skeletal muscle. **(A)** RT-qPCR analysis of miR-451a and miR-144-3p levels (fold) in old (18.5 months) relative to young (2 months) mouse skeletal muscle (gastrocnemius). **(B)** RT-qPCR analysis of *Sparc* mRNA levels in old relative to young mouse skeletal muscle tissues. **(C)** Proposed model of miR-451 influence on SPARC expression as a function of aging. Higher expression of miR-451 in old skeletal muscle can inhibit SPARC expression. We propose that by inhibiting SPARC expression in old skeletal muscle, miR-451a suppresses myogenesis in old muscle.
(PDF)

S1 Table Oligomers for qPCR amplification used in this study. (XLSX)

S2 Table Microarray analysis of mRNA expression levels in C2C12 cells. (XLSX)

S3 Table Targets of mmu-miR-451a reported in starBase V2.0. (XLSX)
