# Supplementary material for: Loss of miR-451a enhances SPARC production during myogenesis
Source: PLoS One. 2019 Mar 29;14(3):e0214301. doi: 10.1371/journal.pone.0214301 (PMC6440632; doi:10.1371/journal.pone.0214301)
Supplement: S1 Materials and Methods — (DOCX) [file pone.0214301.s001.docx]

**Materials and methods**

**Human myoblast differentiation and RT-qPCR analysis**

Human KM155 myoblasts were cultured in growth medium (equal volume mixture of Hamm’s F10 media with 20% FBS and Promocell Skeletal Muscle Cell Growth Medium) and were maintained at 37°C in a humidified atmosphere with 5% CO_2_. The confluent myoblasts were differentiated for 6 days in differentiating medium (DMEM supplemented with 2% horse serum). Total RNA from cultured cells was isolated using TRIzol (Life Technologies) and RT-qPCR analysis was performed as described in the main text using gene-specific primers for *GAPDH* mRNA (CTCTGCTCCTCCTGTTCGAC, ACGACCAAATCCGTTGACTC) and *SPARC* mRNA (CTTCAGACTGCCCGGAGA, AGGCAAAGGAGAAAGAAGATCC).
