# Supplementary figures and images for: Loss of miR-451a enhances SPARC production during myogenesis
Source: PLoS One. 2019 Mar 29;14(3):e0214301. doi: 10.1371/journal.pone.0214301 (PMC6440632; doi:10.1371/journal.pone.0214301)

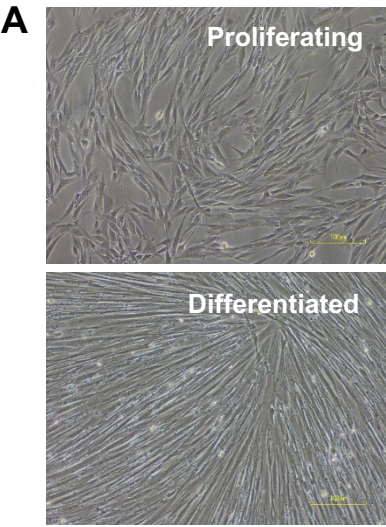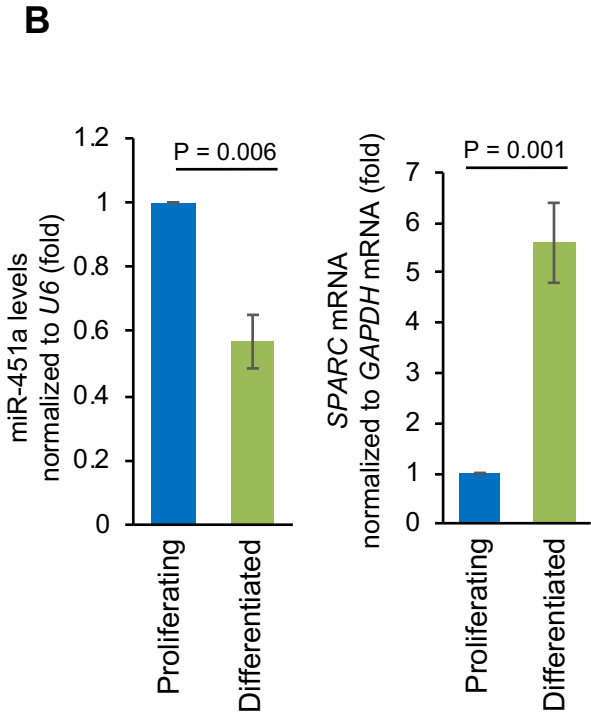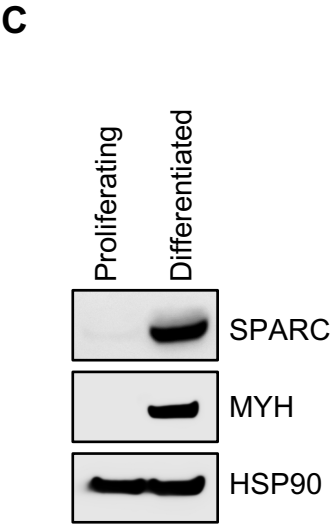

Supplement: S1 Fig — (A) Phase-contrast micrographs of KM155 cells that were cultured in either proliferating medium or cultured for 6 days in differentiating medium at 37°C in a 5% CO2 humidified atmosphere. (B) The cultures described in (A) were used for RT-qPCR analysis of SPARC mRNA and miR-451a in proliferating or differentiated KM155 cultures. Data represent the means ± SEM from 3 or 4 independent experiments. Significance (P) is indicated. (C) Western blot analysis of MYH and SPARC levels in the KM155 cultures described in panel (A). (PDF) [file pone.0214301.s002.pdf]

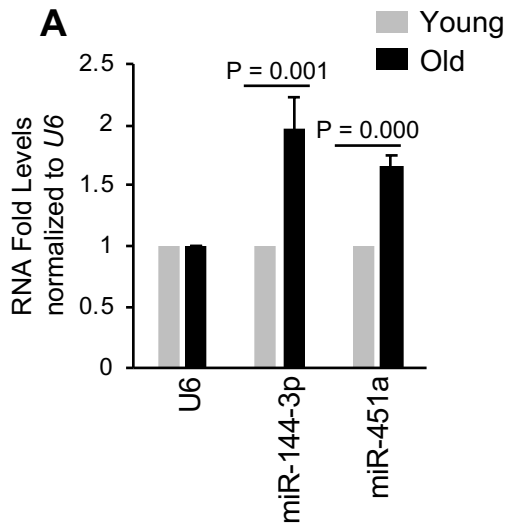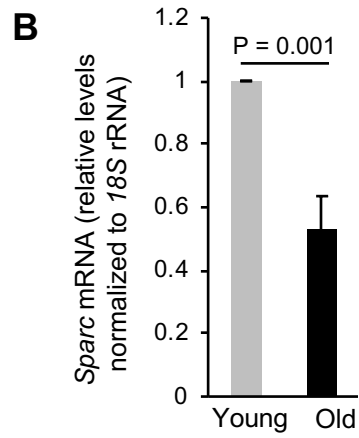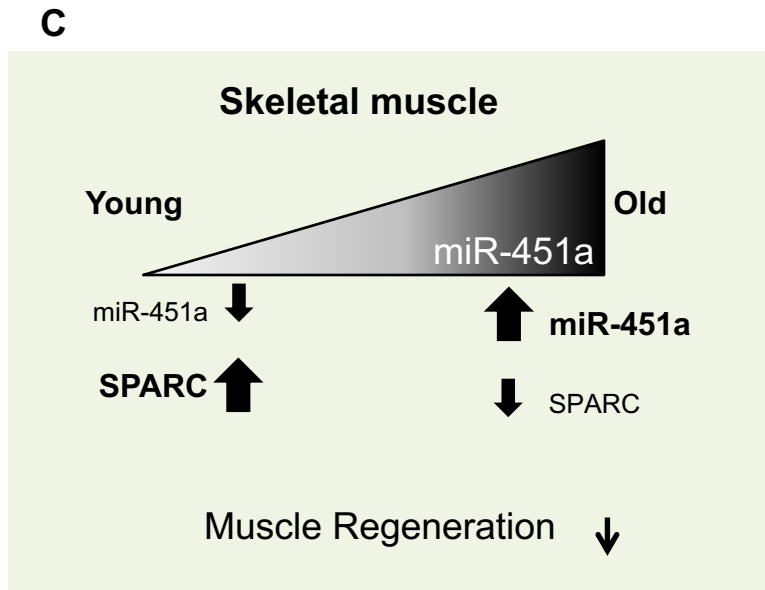

Supplement: S3 Fig — (A) RT-qPCR analysis of miR-451a and miR-144-3p levels (fold) in old (18.5 months) relative to young (2 months) mouse skeletal muscle (gastrocnemius). (B) RT-qPCR analysis of Sparc mRNA levels in old relative to young mouse skeletal muscle tissues. (C) Proposed model of miR-451 influence on SPARC expression as a function of aging. Higher expression of miR-451 in old skeletal muscle can inhibit SPARC expression. We propose that by inhibiting SPARC expression in old skeletal muscle, miR-451a suppresses myogenesis in old muscle. (PDF) [file pone.0214301.s004.pdf]
