# Supplementary material for: Loss of miR-451a enhances SPARC production during myogenesis
Source: PLoS One. 2019 Mar 29;14(3):e0214301. doi: 10.1371/journal.pone.0214301 (PMC6440632; doi:10.1371/journal.pone.0214301)

A

**Sparc:mmu-miR-451a**	
Target Location	chr11:55395438-55395458[-]
Target Name	Sparc
ClipSeq peakCluster	**MHDBR_7195** (AGO_HITS-CLIP_Brain)
ClipSeq ReadNum	**25**

B

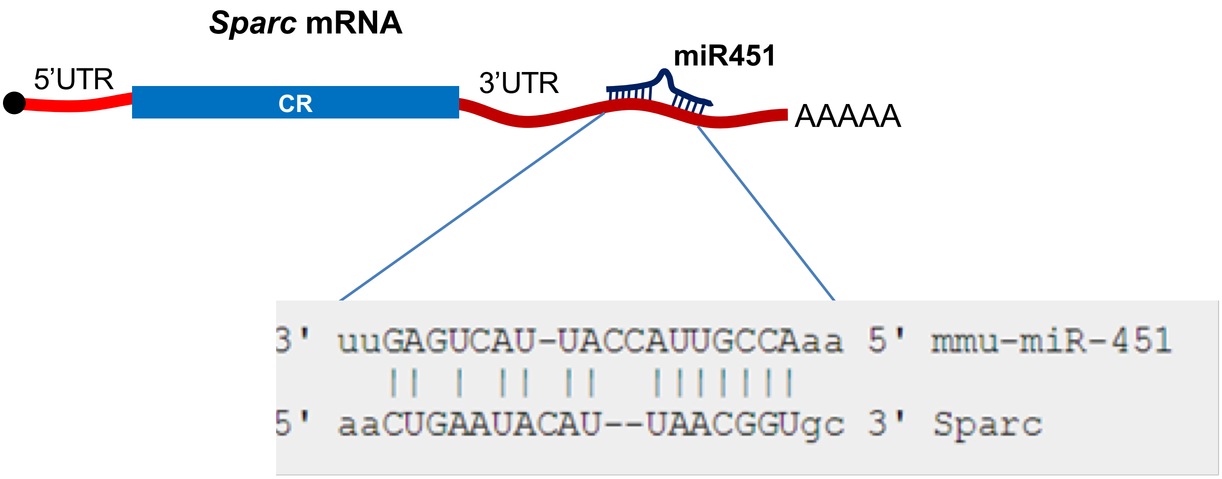

Supplement: S2 Fig — (A) AGO HITS-CLIP data reported by starBase V2.0 (http://starbase.sysu.edu.cn/). (B) Schematic representation of miR-451a target site in the 3’UTR of mouse Sparc mRNA as predicted by microrna.org that uses the miRanda algorithm for target prediction. (PDF) [file pone.0214301.s003.pdf]
